# Genetic Authentication of *Gardenia jasminoides* Ellis var. grandiflora Nakai by Improved RAPD-Derived DNA Markers

**DOI:** 10.3390/molecules201119687

**Published:** 2015-11-10

**Authors:** Zhiqiang Mei, Boxu Zhou, Chunli Wei, Jingliang Cheng, Saber Imani, Hanchun Chen, Junjiang Fu

**Affiliations:** 1 Research Center for Preclinical Medicine, Sichuan Medical University, Luzhou 646000, China; meizhiqiang@scmu.edu.cn (Z.M.); 20140199120007@stu.scmu.edu.cn (B.Z.); weichunli2015@scmu.edu.cn (C.W.); jingliangc@scmu.edu.cn (J.C.); saberimani@scmu.edu.cn (S.I.); 2 State Key Laboratory of Quality Research in Chinese Medicine, Macau University of Science and Technology, Macau (SAR) 999078, China; 3 Chemical Injuries Research Center, Baqiyatallah University of Medical Sciences, Tehran 14359-44711, Iran; 4 Department of Biochemistry, School of Life Sciences & the State Key Laboratory of Medical Genetics, Central South University, Changsha 410013, China; chenhanchun@csu.edu.cn; 5 Judicial Authentication Center, Sichuan Medical University, Luzhou 646000, China

**Keywords:** *G. jasminoides* Ellis var. grandiflora Nakai, *Gardenia jasminoides*, random amplified polymorphic DNA, sequence-characterized amplified region, cloning, genetic authentication

## Abstract

The evergreen shrub, *Gardenia jasminoides* Ellis var. grandiflora Nakai is one of the most popular garden-plants, with significant ornamental importance. Here, we have cloned improved random amplified polymorphic DNA (RAPD) derived fragments into T-vector, and developed sequence-characterized amplified region (SCAR) markers. These markers have been deposited in GenBank database with the accession numbers KP641310, KP641311, KP641312 and KP641313 respectively. The BLAST search of database confirmed the novelty of these markers. The four SCAR markers, namely ZZH11, ZZH31, ZZH41 and ZZH51 can specifically recognize the genetic materials of *G. jasminoides* from other plant species. Moreover, SCAR marker ZZH31 can be used to distinguish *G. jasminoides* Ellis var. grandiflora Nakai from other *G. jasminoides* on the market. Together, this study has developed four stably molecular SCAR markers by improved RAPD-derived DNA markers for the genetic identification and authentication, and for ecological conservation of medicinal and ornamental plant *G. jasminoides*.

## 1. Introduction

*Gardenia jasminoides* Ellis var. grandiflora Nakai (*G. jasminoides* Ellis var. grandiflora Nakai) is an evergreen shrub, commonly used as a garden-plant or house-plant of ornamental importance. *G. jasminoides* Ellis var. grandiflora Nakai is a variety of *Gardenia jasminoides* (*G. jasminoides*) with both ornamental and medicinal importance. Some studies have revealed that active ingredients isolated from different variety of *G. jasminoides* or extracts from this plant, are effective against inflammation, renal failure, metabolic and histological abnormalities in fatty liver disease, fibrosis, thrombosis, acute injuries of lungs, and some types of cancer, viral infections, depressions, and amyloid beta peptide cytotoxicity in Alzheimer’s disease [1,2,3,4,5,6,7,8,9,10,11].

*G. jasminoides* has commonly been found wild growing in several regions of the world, including China, for more than a thousand years. In spite of its ornamental and medicinal importance, very few studies have been conducted on proper genetic identification of *G. jasminoides*. Our previous study reported the genetic variations among *G. jasminoides* Ellis var. grandiflora Nakai collected from geographically different cities in China [12], suggesting this plant species is genetically vulnerable. Therefore, this plant species can likely be confused, if it is not genetically identified and characterized.

Molecular marker technologies are important bio-techniques, which are commonly used for the characterization, identification, and variety authentication of certain species of living organisms. Random amplified polymorphic DNA (RAPD) is a widely used technique, which was introduced three decades ago, and then developed for genetic characterization of organisms [13,14,15,16]. Although RAPD has gained popularity because of its advantages, it also has some disadvantages including low production and poor reproducibility. The sequence characterized amplified region (SCAR) markers are very stable, which are generally derived from the molecular cloning of RAPD fragments [17,18,19]. In our previous study [12], we have genetically characterized *Gardenia jasminoides* Ellis var. grandiflora Nakai by using an improved method RAPD analysis. On the basis of that study, here we have cloned the RAPD fragments, and developed some SCAR markers, which can specifically identify cultivars of *G. jasminoides* at the DNA level.

## 2. Results and Discussion

### 2.1. Results

#### 2.1.1. Cloning of RAPD Amplification Fragments

**Figure 1:**
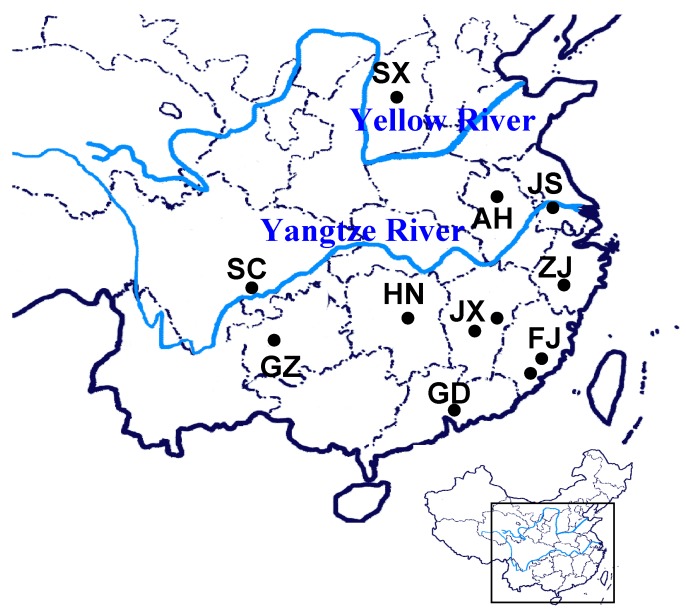
The localities of the samples of *G. jasminoides* Ellis var. grandiflora Nakai and *G. jasminoides* from different regions in China. The spots in dark blue indicate the localities where the samples were collected, the lines in light blue indicate the Yangtze River and Yellow River.

The sources of the *G. jasminoides* Ellis var. grandiflora Nakai accessions used in the RAPD analysis are listed in Table 1. Two RAPD primers SBC-N5 (N5) and SBC-Q12 (Q12) were used for the RAPD amplification in six previous described samples of *G. jasminoides* Ellis var. grandiflora Nakai [12]. The localities of the samples of *G. jasminoides* Ellis var. grandiflora Nakai and *G. jasminoides* are shown in Figure 1.

**Table 1:** Sources of RAPD-SCAR samples for cultivars of *G. jasminoides* Ellis var. grandiflora Nakai and *G. jasminoide*.

Sample	Species	Source	No.
JX	*G. jasminoides* Ellis var. grandiflora Nakai	Shangrao, Jiangxi	1
FJ	*G. jasminoides* Ellis var. grandiflora Nakai	Fuzhou, Fujian	2
GZ	*G. jasminoides* Ellis var. grandiflora Nakai	Anshun, Guizhou	3
SC	*G. jasminoides* Ellis var. grandiflora Nakai	Luzhou, Sichuan	4
HN	*G. jasminoides* Ellis var. grandiflora Nakai	Changsha, Hunan	5
ZJ	*G. jasminoides* Ellis var. grandiflora Nakai	Lishui, Zhejiang	6
SC2	*G. jasminoides* (*G. jasminoides* J. Ellis)	Luzhou, Sichuan	7
GD	*G. jasminoides* Ellis var. grandiflora Nakai	Shenzhen, Guangdong	8
FJ2	*G. jasminoides* Ellis var. grandiflora Nakai	Quanzhou, Fujian	9
HN2	*G. jasminoides* Ellis var. grandiflora Nakai	Changsha, Hunan	10
AH	*G. jasminoides* Ellis var. grandiflora Nakai	Huainan, Aihui	11
JX2	*G. jasminoides* (*G. jasminoides* J. Ellis)	Fuzhou, Jiangxi	12
JS	*G. jasminoides* (*G. jasminoides* J. Ellis)	Nanjing, Jiangsu	13
HN3	*G. jasminoides* (*G. jasminoides* J. Ellis)	Changsha, Hunan	14
SX	*G. jasminoides* (*G. jasminoides* J. Ellis)	Taiyuan, Shanxi	15

**Figure 2:**
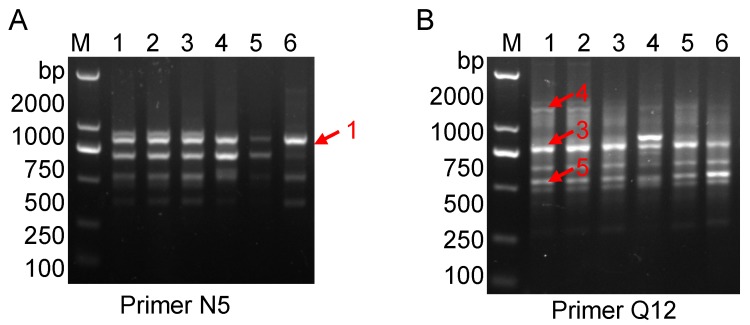
Amplification and recovery of improved random amplified polymorphic DNA (RAPD) fragments. Improve RAPD amplification from DNA samples of *G. jasminoides* Ellis var. grandiflora Nakai which listed in Table 1 (Nos. 1~6). (**A**) The primer SBC-N5 (N5); (**B**) The primers SBC-Q12 (Q12). The red arrows indicate bands before cut. *Lane* M indicates the DNA molecular weight marker DL2000 with the fragment size (bp) 2000, 1000, 750, 500, 250, 100.

**Figure 3:**
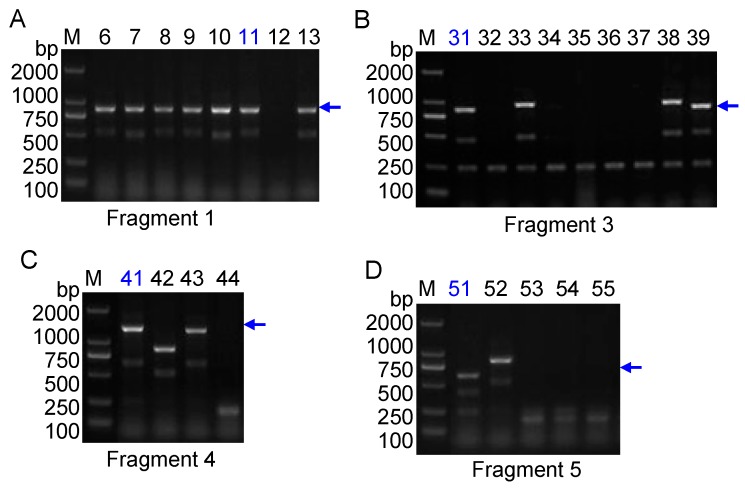
DNA cloning and identification of positive clones. (**A**) Clone identification of RAPD fragment 1. *Lanes* 6~12 indicate different clones; (**B**) Clone identification of RAPD fragment 3. *Lanes* 31~39 indicate different clones; (**C**) Clones identification of RAPD fragment 4. *Lanes* 41~44 indicate different clones; (**D**) Clone identification of RAPD fragment 5. *Lanes* 51~55 indicate different clones. The blue arrows indicate positive PCR products. Clones ZZH11, ZZH31, ZZH41 and ZZH51 in blue colors were sequencing. The blue arrows indicate expected PCR bands in size of different clones. *Lane* M indicates the DNA molecular weight marker DL2000 with the fragment size (bp).

The improved RAPD amplification results are shown in Figure 2, where red arrows indicate the bands labeled with by primer N5 (Figure 2A) and Q12 (Figure 2B). The arrow-indicated bands were cut from an agarose gel and further purified, and then ligated to T-vector by TA cloning. The blue/white screening method in LB agar plate was adopted firstly to screen the positive clones (data not shown). The white clones were then identified by polymerase chain reaction (PCR) amplification using SP6/T7 primer pair that is mentioned in the Experimental section. In Figure 3A the clone ZZH11 is shown in lane 7 as an expected inserted DNA-fragment with ~800 bp in size, whereas the clones ZZH31, ZZH41 and ZZH51 are shown in Figure 3B–D, as three inserted DNA-fragments with right length in sizes, respectively. Clones ZZH11, ZZH31, ZZH41 and ZZH51 were finally selected for Sanger sequencing.

#### 2.1.2. Sequencing and Characterization of *G. jasminoides* Ellis var. grandiflora Nakai Specific RAPD Fragments

**Figure 4:**
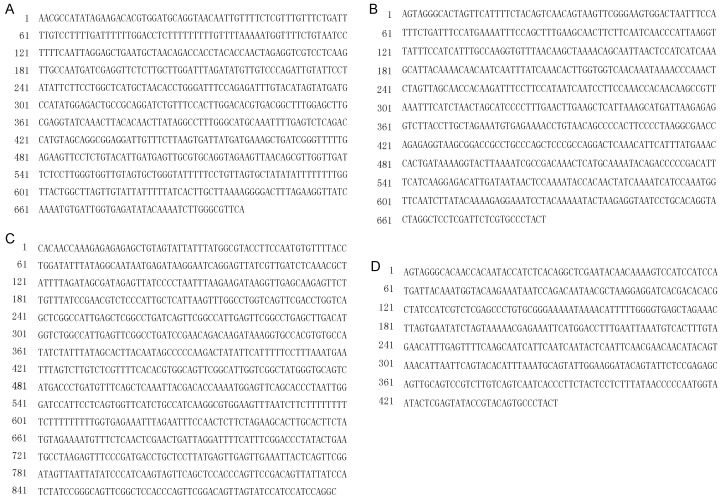
The cloned sequences information by Sanger-sequencing. (**A**) The sequences of clone ZZH11; (**B**) The sequences of clone ZZH31; (**C**) The sequences of clone ZZH41; (**D**) The sequences of clone ZZH51.

After sequencing of the above mentioned four RAPD fragments clones of *G. jasminoides*, BLAST searches of the nucleotide sequences in GenBank database were performed and indicated that these clones have no significant identity to that of any species. The sequencing results revealed that clone ZZH11, consisting of 700 nucleotides, was deposited into GenBank with accession number KP641310 (Figure 4A); cloneZZH31, consisting of 699 nucleotides, was deposited into GenBank with accession number KP641311 (Figure 4B); clone ZZH41, consisting of 942 nucleotides, was deposited into GenBank with accession number KP641312 (Figure 4C); and clone ZZH51, consisting of 457 nucleotides, was deposited into GenBank with accession number KP641313.

#### 2.1.3. Development of Specific SCAR Markers for *G. jasminoides* Ellis var. grandiflora Nakai, and Analysis of the PCR Amplicons at Different Species

**Figure 5:**
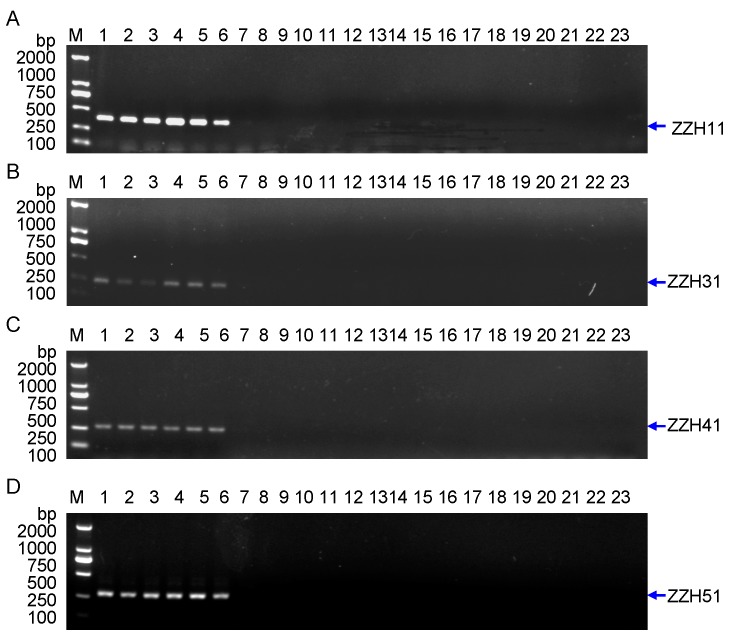
Development of stable RAPD-sequence-characterized amplified region (SCAR) markers for ZZH11, ZZH31, ZZH41 and ZZH51. (**A**) A SCAR marker ZZH11; (**B**) A SCAR marker ZZH31; (**C**) A SCAR marker ZZH41; (**D**) A SCAR marker ZZH51. *Lanes* 1~6 indicate the different samples of *G. jasminoides* Ellis var. grandiflora Nakai listed in Table 1. *Lanes* 7 and 8 are two samples of *Lonicera japonica* from Hunan and Hubei; *Lane* 9 is one sample of *Penthorum chinense*; *Lane* 10 is one sample of *Ganoderma lucidum*; *Lanes* 11 and 12 are two samples of *Litchi chinesis* from Guangdong and Sichuan; *Lanes* 13 and 14 are two samples of *Dimocarpus Longan* from Fujian and Sichuan; *Lane* 15 is one sample of *Dimocarpus confinis* from Guangxi; *Lanes* 16 and 17 are two samples of *Ginkgo Biloba* from Sichuan and Hunan; *Lanes* 18 and 19 are two samples of *Angelica sinensis* from Sichuan and Gansu; *Lane* 20 is one sample of *Gastrodia elata*; *Lanes* 21 and 22 are two samples of *Canarium album* Guangdong and Sichuan; *Lane* 23 is negative control without DNA. The blue arrows indicate expected PCR products in size. *Lane* M indicates the DNA molecular weight marker DL2000.

In this study, we designed and synthesized four pairs of primers (for ZZH11, ZZH31, ZZH41 and ZZH51) to generate more stable *G. jasminoides* Ellis var. grandiflora Nakai-specific diagnostic SCAR markers based on our cloned sequences (Figure 4). The primers used in the RAPD analysis are listed in Table 2. The designed SCAR primer pairs were used to amplify the genomic DNA collected from 22 samples to test amplification species-specificity. The PCR amplification results are shown in Figure 5. The PCR results by SCAR markers ZZH11, ZZH31, ZZH41 and ZZH51 (Figure 5) indicated that the PCR products with expected size were observed only in samples of *G. jasminoides*, and no amplification in other species we tested. This indicates that the SCAR marker ZZH11, ZZH31, ZZH41 and ZZH51 are specific for *G. jasminoides* Ellis var. grandiflora Nakai. The lack of this specific amplicons in the samples from other species indicates the efficacy of this marker in distinguishing the samples of *G. jasminoides* Ellis var. grandiflora Nakai from other species. Negative controls without DNA template did not show any PCR product (data not shown). Therefore, we confirm that *G. jasminoides*-specific SCAR markers were successfully developed, which can be used for the authentication of these plants.

**Table 2:** Sequences of SCAR primers, PCR condition and product size (bp).

SCAR	5′-primer	Sequence (5′–3′)	3′-primer	Sequence (5′–3′)	Size	Tm (°C)
ZZH11	ZZH11-L	TGCCAATGATCGAGGTTCTC	ZZH11-R	GCACTACAACCACCCAAGGA	381	60
ZZH31	ZZH31-L	TTCCAAACCACAACAAGCCGT	ZZH31-R	GCATGAGTTTGTCGGCGATTT	245	64
ZZH41	ZZH41-L	ATGGCGTACCTTCCAATGTG	ZZH41-R	AATGGCCAGACCATGTCAAG	279	60
ZZH51	ZZH51-L	ACGCTAAGGAGGATCACGACA	ZZH51-R	ACTGACAAGACGGACTGCAAC	291	60

**Figure 6:**
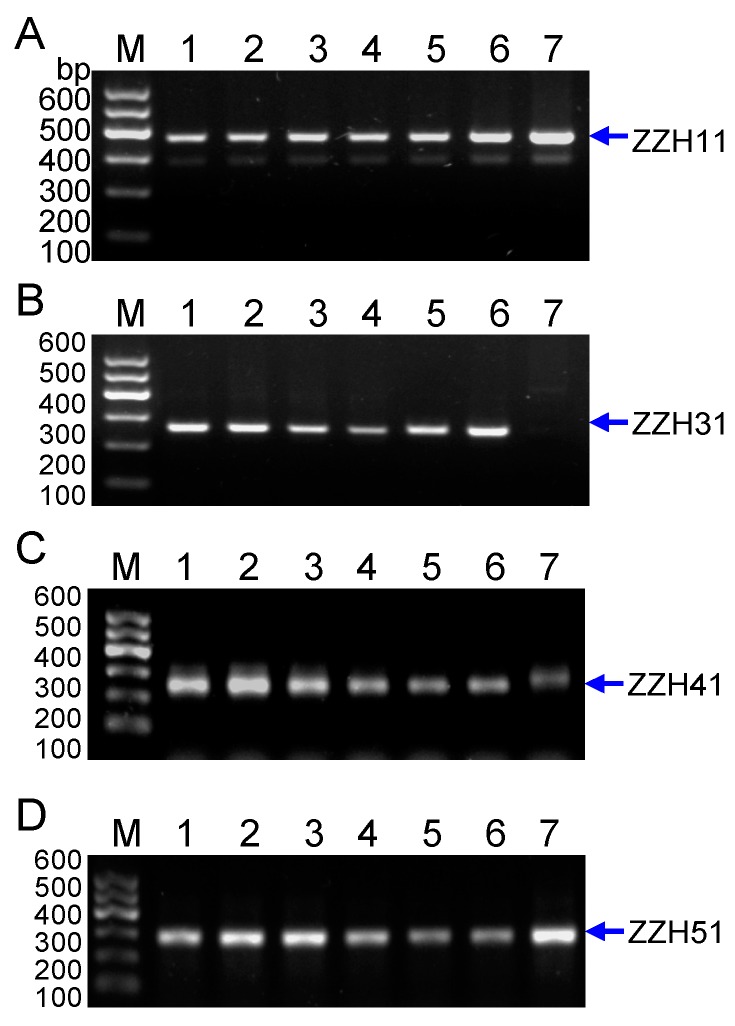
Genetic authentication of *Gardenia jasminoides* based on ZZH11 (**A**); ZZH31 (**B**); ZZH41 (**C**) and ZZH51 (**D**) SCAR markers. *Lanes* 1–6 are the DNA samples of *G. jasminoides* Ellis var. grandiflora Nakai from Jiangxi, Fujian, Guizhou, Sichuan, Hunan and Zhejiang respectively (Table 1). *Lane* 7 is a sample of *G. jasminoides* from Luzhou in Sichuan (No. 7 in Table 1). *Lane* M indicates the DNA molecular weight marker DL600 with the fragment size (bp).

**Figure 7:**
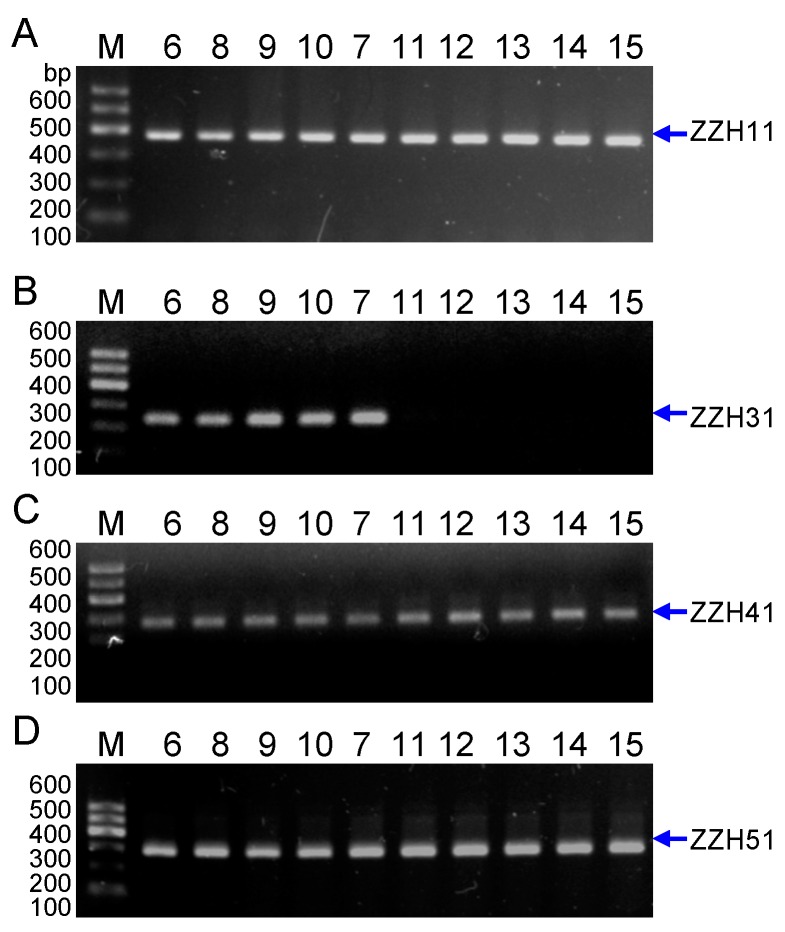
Genetic authentication of more *Gardenia jasminoides* sample based on ZZH11 (**A**); ZZH31 (**B**); ZZH41 (**C**) and ZZH51 (**D**) SCAR markers. *Lanes* 6, 8, 9, 10 and 11 are the DNA samples of *G. jasminoides* Ellis var. grandiflora Nakai from Zhejiang, Guangdong, Fujian, Hunan and Anhui respectively (Table 1, Nos. 6, 8, 9, 10 and 11). *Lane* 7, 12, 13, 14 and 15 are samples of *G. jasminoides* from Luzhou in Sichuan, Jiangsu, Jiangxi, Hunan and Shanxi respectively (Table 1, Nos., 7, 12, 13, 14 and 15). *Lane* M indicates the DNA molecular weight marker DL600 with the fragment size (bp).

Then, to further test whether these four markers can be amplified in the sample of *G. jasminoides* we collected in the market from Luzhou of Sichuan (Table 1), the results in the Figure 6 showed that only SCAR marker ZZH31 were amplified in the sample from *G. jasminoides* Ellis var. grandiflora Nakai, not from *G. jasminoides*, whereas other three SCAR markers ZZH11, ZZH41and ZZH51 were amplified in the samples of both *G. jasminoides* Ellis var. grandiflora Nakai and *G. jasminoides*. Again, four *G. jasminoides* samples and four *G. jasminoides* Ellis var. grandiflora Nakai samples were collected from different localities (Table 1, Nos. 8~15), all four makers can be amplified in samples of *G. jasminoides* and *G. jasminoides* Ellis var. grandiflora Nakai, except that marker ZZH31 can’t amplified in the samples of *G. jasminoides*, further supporting that SCAR marker ZZH31 is specific for *G. jasminoides* Ellis var. grandiflora Nakai (Figure 7).

### 2.2. Discussion

This investigation aimed to improve the authentication process of *G. jasminoides* Ellis var. grandiflora Nakai using a RAPD-derived SCAR molecular method. Authentication and characterization of any living organisms has been revolutionized with the development of molecular marker technologies. Over the last three decades, numerous molecular marker techniques have been developed, including RAPD, amplified fragment length polymorphism (AFLP) analysis, simple sequence repeat (SSR) analysis, inter-simple sequence repeat (ISSR) analysis and *etc.* [13,14,20,21]. It has been reported that the genetic identification and authentication of any species or cultivars become more specific and stable, when another bio-technique SCAR is combined with RAPD [16,18,19,22,23,24,25].

To develop the SCAR markers, in this study, we have at first collected six cultivars of *G. jasminoides* Ellis var. grandiflora Nakai, from different geographically isolated regions of China. The DNA materials of these plant leaves were extracted and amplified by an improved RAPD, which revealed the significant genetic variations among the cultivars [12]. We have then focused on the development of SCAR markers specific to *G. jasminoides* Ellis var. grandiflora Nakai, which can identify this species or cultivars from others. The RAPD fragments were cloned and then four SCAR markers ZZH11, ZZH31, ZZH41 and ZZH51 were developed, which are specific to all of the cultivars of *G. jasminoides* Ellis var. grandiflora Nakai species used in this study. Notably, DNA material from other species was not recognized by these four markers (Figure 5). Further investigation demonstrated that only SCAR marker ZZH31 were amplified in the sample from *G. jasminoides* Ellis var. grandiflora Nakai, not from *G. jasminoides* when tested by collecting five samples from different localities, whereas other three SCAR markers ZZH11, ZZH41 and ZZH51 were amplified in the samples of both *G. jasminoides* Ellis var. grandiflora Nakai and *G. jasminoides* samples. In line with previous studies, this clearly indicates that these three SCAR markers ZZH11, ZZH41 and ZZH51 are specific to *Gardenia* family, whereas SCAR marker ZZH31 is strictly specific to *G. jasminoides* Ellis var. grandiflora Nakai cultivars. Thus, we could use SCAR marker ZZH31 to distinguish *G. jasminoides* Ellis var. grandiflora Nakai from *G. jasminoides* in our markets (Figure 6 and Figure 7).

Expectedly, in GenBank database, the BLAST searches of these four nucleotide sequences did not show significant identity to that of any species. This indicates that ZZH11, ZZH31, ZZH41 and ZZH51 are the novel biomarkers for the identification and authentication of *G. jasminoides*, particularly *G. jasminoides* Ellis var. grandiflora Nakai. In some previous studies, RAPD-SCAR marker techniques have been successfully developed for genetic identification of some other plants, like *Lonicera japonica*, *Dimocarpus longan*, and other *etc.* [18,19,21,22,23,24,25,26], however, as our best knowledge, this is the first study, which developed molecular SCAR markers for the authentication of *G. jasminoides* species. Although microsatellite markers for the studying species *G. jasminoides* have been developed very recently [27,28,29]. The results highlighted that development of these SCAR markers will be helpful for genetic and ecological preservation and conservation of this plant. We suggest that RAPD-SCAR technology could be useful for the identification and characterization of different Chinese herbal materials in the plant pharmaceutical industry or of ornamental importance.

## 3. Experimental Section

### 3.1. DNA Extraction from G. jasminoides

Firstly, six fresh young leaves of different cultivars of *G. jasminoides* Ellis var. grandiflora Nakai which was previous described [12], five more *G. jasminoides* Ellis var. grandiflora Nakai and five *G. jasminoides* were collected (Table 1, Figure 1). All the samples of *G. jasminoides* Ellis var. grandiflora Nakai were collected from gardens from different localities and all the samples of *G. jasminoides* were purchased in the pharmacy stores which indicated their originations clearly. The samples of *G. jasminoides* are dried fruits. Plant specimens were identified by the authors and all voucher specimens have been deposited at the Medicinal Botanical Association of Zhongshan Mountain (MBAZM), Sichuan Medical University. Then the plant genomic DNA materials were extracted as previously described slightly modified cetyltrimethylammonium bromide (CTAB) method [29]. DNA was then diluted with 1 × TE buffer to make the final concentration of 10 ng/μL, and stored at −20 °C until usage.

### 3.2. Amplification of DNA by Improved RAPD

DNA materials of different cultivars of *G. jasminoides* Ellis var. grandiflora Nakai samples were amplified with primers SBC-N5 and SBS-Q12 using Tiangen Biotech reagents (Beijing, China) according to the manipulation protocol. A total 10 μL PCR reaction system consisted of 5 μL 2 × Taq PCR MasterMix, 1 μL 2.5 μM primer, 1.5 μL genomic DNA, and 2.5 μL ddH_2_O. Amplification reactions were performed in an Applied Biosystems Veriti^®^ 96-Well Thermal Cycler (Life Technology, Carlsbad, CA, USA) using the following program: initial denaturation at 95 °C for 90 s, followed by 40 cycles of denaturation at 94 °C for 40 s, annealing at 36 °C with the RAMP rate from annealing to extension adjusted to 0.125 °C/s (5% ramp rate) for 60 s, extension at 72 °C for 90 s, and a final extension step at 72 °C for 5 min [12]. PCR products were loaded into a 1.5% agarose gel for electrophoresis, along with 1 kb or 100 bp DNA ladder markers (Tiangen Biotech). Measurements were repeated three times in each well.

### 3.3. Cloning, Identification and Sequencing of DNA Fragments

The DNA Cloning, identification and sequencing were described previously [16,18]. Briefly, four different bright bands were excised from the agarose gel, and purified by using TIANgel Mini Purification Kit (DP209, Tiangen Biotech). The purified DNA fragments were ligated into pGM-T vector (No. VT202) (Tiangen Biotech), and transformed into DH5α *E. coli* competent cells. The recombinant clones were seeded overnight at 37 °C on LB agar plates, containing 100 μg/μL of ampicillin, 40 mg of X-gal and 160 μg of IPTG and kept at 37 °C for overnight. The white colonies were screened out by blue white screening method. The presence of right insert was verified by PCR by using T7/SP6 primer pairs (T7 primer: 5′-TAATACGACTCACTATAGGG-3′, SP6 primer: 5′-ATTTAGGTGACACTATAGAA-3′), then run on a 1% agarose gel electrophoresis [16,18]. The sequencing of the positive clones was performed by Sanger di-deoxy sequencing from Beijing ZiXi Biological Technology Co., Ltd. (Bejing, China) using ABI3500 sequencer (Applied Biosystems Inc., Foster City, CA, USA).

### 3.4. Bioinformatic Analysis by Online Program BLAST

To remove the vector sequences and verify whether the sequences of cloned RAPD fragments are novel, the online program BLAST [30] was used for the homology search of sequenced DNA from different species in GenBank database. Database searches of sequence homology were performed using the program BlastN set to general parameters (expect threshold was 10).

### 3.5. Design of SCAR Primers

The nucleotide sequence of each of the cloned RAPD fragment was used to design pairs of SCAR primers using online program Primer 3 v.0.4.0 [31]. The sequences of each primer, optimized PCR condition and amplification length are shown in Table 2.

### 3.6. Development SCAR Markers and SCAR Analysis

To develop SCAR markers, the PCR amplification was performed by using DNA template from 11 different species, including six cultivars of *G. jasminoides* Ellis var. grandiflora Nakai and another 10 medicinal plant species (16 samples in total) [12,13,14,15,16,18]. The content of 10 μL PCR reaction system was as follows: 5 μL 2 × Taq PCR MasterMix, 1 μL of 2.5 μM each pair of SCAR primers, and 1 μL genomic DNA (10 ng), with the remaining volumes filled by ddH_2_O. PCR was performed in an Applied Biosystems Veriti^®^ 96-Well Thermal Cycler with an initial pre-denaturation for 90 s at 95 °C followed by 27~35 cycles of denaturation at 94 °C for 40 s, annealing at 60 °C or 64 °C for 30 s, and extension at 72 °C for 40 s. The final extension step was performed at 72 °C for 5 min. The amplified PCR products were separated by electrophoresis on a 1.8% agarose gel in 1 × TAE buffer. Gels were then visualized by 0.5 μg/mL ethidium bromide staining and the images were documented using the ChemiDoc XRS (Bio-Rad, Hercules, CA, USA) [29].

## 4. Conclusions

We have cloned and sequenced four improved RAPD derived fragments which were deposited in the GenBank database with the accession numbers KP641310, KP641311, KP641312 and KP641313 respectively, and developed SCAR markers, named ZZH11, ZZH31, ZZH41 and ZZH51 which can specifically recognize the genetic materials of *G. jasminoides* from other plant species. Moreover, SCAR marker ZZH31 can be used to distinguish *G. jasminoides* Ellis var. grandiflora Nakai from *G. jasminoides* at the DNA level. Thus, this study has developed four stably molecular SCAR markers by improved RAPD-derived DNA markers for the genetic identification and authentication, and for ecological conservation of medicinal and ornamental plant *G. jasminoides*. We suggest that RAPD-SCAR technology could be useful for the identification and characterization of different Chinese herbal materials in the plant pharmaceutical industry or of ornamental importance.
